# Methylchloroisothiazolinone and Methylisothiazolinone 10 Years After Regulation: A Case Story of Liquid Soaps and Shampoos

**DOI:** 10.1111/cod.70202

**Published:** 2026-06-16

**Authors:** Mathias Krogh Pedersen, Jakob Ferløv Baselius Schwensen, Jose Hernán Alfonso, Christel Søgaard Kirkeby, Stine Müller, Steen Mollerup, Gianluca Selvestrel, Christina Rudén, Martin F. Wilks, Jeanne Duus Johansen

**Affiliations:** ^1^ The National Allergy Research Centre (Videncenter for Allergi), Department of Allergy, Dermatology and Venerology Copenhagen University Hospital Herlev‐Gentofte, Gentofte Hospital Hellerup Denmark; ^2^ Faculty of Health and Medical Sciences, Department of Clinical Medicine University of Copenhagen Copenhagen Denmark; ^3^ Section of Occupational Toxicology National Institute of Occupational Health Oslo Norway; ^4^ Department of Dermatology and Venereology Oslo University Hospital Oslo Norway; ^5^ The Danish Consumer Council THINK Chemicals (Forbrugerrådet TAENK Kemi) Copenhagen Denmark; ^6^ Environmental Sustainability for Industrial and Health Systems Unit, Environmental Health Department Istituto di Ricerche Farmacologiche Mario Negri IRCCS Milano Italy; ^7^ Division of Toxicology Wageningen University & Research Wageningen the Netherlands; ^8^ Department of Environmental Science Stockholm University Stockholm Sweden; ^9^ Department of Pharmaceutical Sciences University of Basel Basel Switzerland

**Keywords:** contact allergy, cosmetics, methylchloroisothiazolinone, methylisothiazolinone

## Abstract

**Background:**

Contact allergy to methylchloroisothiazolinone (MCI) and methylisothiazolinone (MI) remains common in the EU despite regulation.

**Objectives:**

To assess the extent of use of MCI/MI and MI in cosmetic products on the Danish market, identify product categories involved and evaluate compliance of rinse‐off products with the 15 ppm limit.

**Materials and Methods:**

Products listing MCI/MI or MI were identified through Kemiluppen, a Danish database of marketed cosmetics. Thirty products (16 shampoos and 14 liquid soaps) were examined using liquid chromatography–tandem mass spectrometry: 25 labelled as containing MCI/MI or MI, 5 unlabelled.

**Results:**

In total, 257 cosmetic products containing MCI/MI or MI were identified, primarily shampoos (127), conditioners (98) and liquid soaps (8). Chemical analysis showed that 2 of 25 labelled products (8%) exceeded the 15 ppm limit, and 4 products did not meet the required 3:1 MCI/MI ratio.

**Conclusion:**

MCI/MI is used infrequently in cosmetic products. All, except two products complied with the limit in the regulation. Given the continued occurrence of contact allergy, this indicates that the limit (15 ppm) may be too high to be sufficiently protective. Further investigation into MCI/MI use in cosmetic products is warranted, alongside more proactive risk assessment and management strategies.

## Graphical abstract

MCI/MI and MI contact allergy remain persistent. In this analysis of the Danish market, 257 products containing MCI/MI or MI were identified, and 30 products were analysed. Two exceeded regulatory limits, while most complied, suggesting current limits may require further investigation. Created in BioRender. Pedersen, M. K. (2026) https://BioRender.com/p1jr69x.

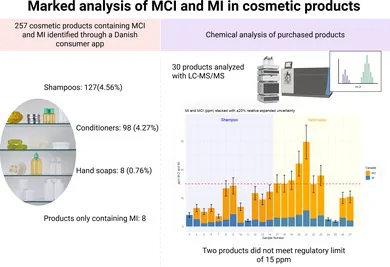

## 1 Introduction

Preservatives are necessary to prevent microbial spoilage of water‐based chemical substances. Isothiazolinones are a group of preservatives that are highly effective for this purpose; however, their use is associated with an inherent risk of contact allergy. A combination of methylchloroisothiazolinone (MCI) and methylisothiazolinone (MI) marketed in a 3:1 ratio has been widely used since the 1980s as a preservative in cosmetic, household and industrial products [1]. The use of MCI/MI led to numerous cases of contact allergy in the European population, and in 1989 the permitted concentration of this mixture was restricted to 15 ppm in cosmetic products [2]. Following this restriction, the prevalence of contact allergy to MCI/MI stabilised during the 1990s.

Around the turn of the millennium, when the patent for the MCI/MI 3:1 mixture (marketed as Kathon) expired, initiatives were taken to use MI as a standalone preservative. Following the industry's premarket risk assessment, MI was introduced as a standalone preservative in cosmetic products in the European Union, with a maximum permitted concentration of 100 ppm [3]. This was soon followed by a rapid increase in cases with newly diagnosed contact allergy to MI, particularly associated with cosmetic products, wet wipes and water‐based paints [4, 5]. In the following years, awareness increased among multiple stakeholders—including regulators—supported by published surveillance data on MI contact allergy from dermatology departments across Europe. Consequently, following an opinion by the Scientific Committee on Consumer Safety (SCCS) in 2013, the use of MCI/MI and MI was banned in leave‐on cosmetic products [6]. Subsequently, the use of MI in rinse‐off cosmetic products was restricted to 15 ppm under the Cosmetic Regulation, corresponding to MCI/MI (Annex V, Arts. 39 and 57) [7]. In this context, it was emphasized that MCI/MI must only be used in a 3:1 ratio and that MCI/MI as a mixture and MI must not be used together.

Studies have shown a continued declining trend of MI contact allergy in the EU since these regulatory measures. In 2022, a multicentre study including ten dermatology departments across eight European countries reported a decrease in MI contact allergy prevalence from 6% to 2.9% [8]. The same study demonstrated that a substantial proportion of clinically relevant cases were attributable to rinse‐off cosmetic products [8]. Despite this recent decline, MI and MCI/MI remain prevalent allergens of clinical relevance. Therefore, we aimed to determine whether MCI/MI and MI were still present in cosmetic products on the Danish market, to identify the product categories in which they occurred, and to assess whether rinse‐off products complied with current cosmetic regulatory requirements.

## 2 Materials and Methods

### 2.1 Selection of Products

**FIGURE 1:**
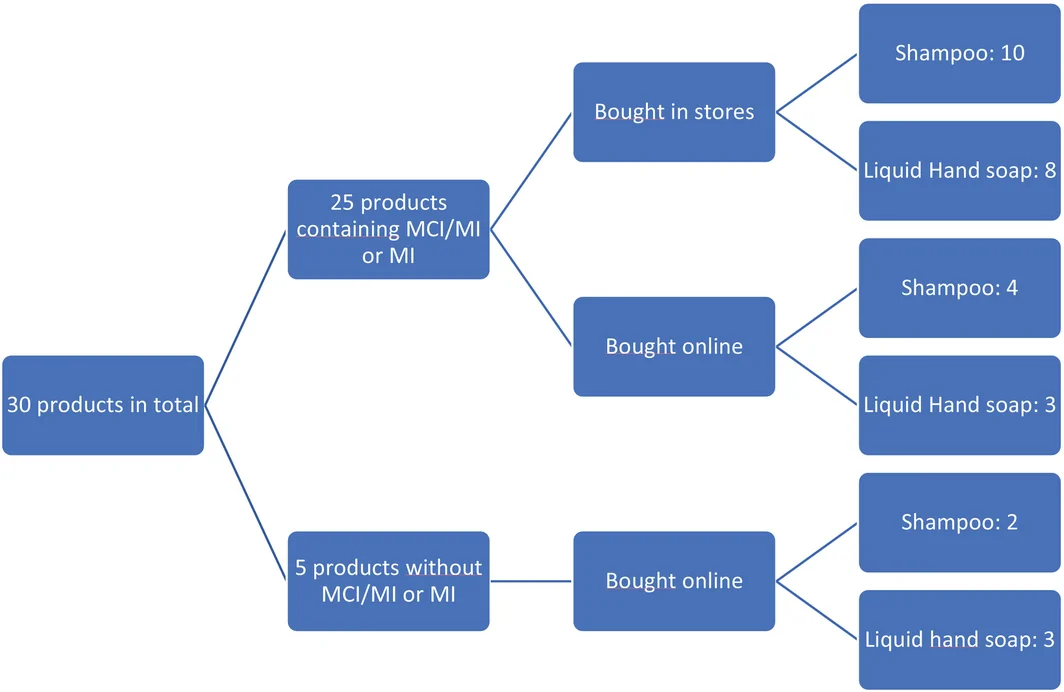
Summary of samples purchased in physical stores in Copenhagen and online. The five products listed as without MCI/MI or MI are products that had no declared MCI/MI or MI content according to the physical ingredients list.

This study utilised data obtained from the ‘Kemiluppen’ a smartphone application with information on cosmetics and detergents on the Danish market, which is a smartphone application developed and managed by the Danish Consumer Council, that guides on problematic chemicals in cosmetics/personal care and detergents. This application has existed since 2015 and contains information on 19.599 current products and in total 20 million product scans have been made (as reported on 21 August 2024) [9]. From this application, we obtained data on all cosmetic and personal care products labelled as containing MI (assuming that all products with MCI also contained MI, thereby also obtaining MCI/MI containing products) and the total number of products of each product type under the category cosmetic and personal care products. Only products currently on the market were searched for. The data obtained was extracted in an excel file with following information: Product category, brand, model and the ingredients list as declared on the product label (reported using INCI nomenclature). The data was extracted the 14th of May 2025, and no duplicates of barcodes were found. From the data obtained, a percentage of products in the product categories containing MCI/MI or MI was calculated. Based on frequency of products labelled with MCI/MI or MI and consideration of expected use patterns from the SCCS notes of Guidance [10], two product categories, shampoo and liquid hand soap, were chosen for chemical analysis. Shampoo was chosen for further analysis since its use is more frequent than conditioner. Liquid hand soap was chosen due to its overall frequent use in private and occupational life. The hand soaps and shampoos were bought based on availability and were not necessarily present in the app Kemiluppen. The products were bought in nonprofessional stores, primarily grocery stores and retail stores, with a price range of 8–139 DKK (approximately 1–19 €). In total 30 products, 16 shampoos and 14 hand soaps (Figure 1), were purchased and analysed.

### 2.2 Chemical Analysis

All products were analysed for MCI and MI content by liquid chromatography coupled tandem mass spectrometry (LC‐MS/MS) by Medico [11]. The sample preparation has been described by Baranowska I. and Wojciechowska I. [12]. One gramme of the samples and 6 mL of methanol were sonicated in a 10 mL graduated flask. Afterwards, methanol was added so the total volume made up 10 mL. Subsequently, the flask was mixed and the sample was filtered through a 0.20 μm filter.

Liquid chromatography was performed following the procedure described by Thermo Fisher's Scientific: Antimicrobial Agents in Hand Soap Determined Using a Thermo Scientific Acclaim RSLC PolarAdvantage (PA) Column (Application note, 2009) [13]. The MS/MS detection electrospray was used in positive mode and selected reaction monitoring values were m/z 115.9 to > 70.9 and 100.9 for MI and m/z 149.8 to > 86.9 and 114.9 for MCI.

The limit of quantification (LOQ) was estimated as three times the limit of detection (LOD), corresponding to 3 ppm. Calibration was performed in the range of 0–10 μg/mL in methanol. Matrix effects were addressed using an isotopically labelled internal standard (D3‐MI). Method validation parameters included a repeatability of 3%, reproducibility of 8% and a recovery of 95%.

The analytical laboratory did not perform a formal assessment of compliance with the regulatory 15 ppm limit or the required 3:1 MCI:MI ratio. Accordingly, any interpretation of these aspects in the present study is based on the reported measured concentrations, taking the stated measurement uncertainty into consideration.

### 2.3 Descriptive Statistics

The frequency of cosmetic and personal care products containing MCI/MI and/or MI was calculated using Excel. The concentrations of MCI/MI and/or MI of the 30 cosmetic and personal care products were analysed and set in perspective to the current legislation restricting the use of MI to 15 ppm in rinse‐off cosmetic and personal care products and graphical representations were made in RStudio [14]. The range of possible ratios between MCI and MI was calculated from the LC‐MS/MS analysis using the measured values of MCI (±20%) and MI (±20%) and applying the uncertainty on the denominator and the numerator.

Concerning the LC‐MS/MS results, previous spiking experiments have shown the relative combined standard uncertainty to be 10% corresponding to an approximate 68% confidence interval for the measurements. The relative expanded uncertainty with a coverage factor of 2 (95% confidence interval) is therefore calculated to be ±20% [15].

## 3 Results

From Kemiluppen, a total of 10 263 products were listed within categories containing MCI/MI and MI products (the 10 product categories shown in Table 1). In total, 257 products contained MCI/MI (249 products) or MI (8 products), primarily found in shampoos (127 products) and conditioners (98 products), followed by liquid hand soaps with 8 products (Table 1). Other product categories with more than one product included cleansing/makeup remover/wash (6 products), hair dye (5 products) and body wash (7 products). In total, 4.56% of shampoo products and 0.76% of scanned liquid hand soaps contained MCI/MI or MI (Table 1).

**TABLE 1:** Frequencies of products labelled as containing MCI and/or MI for each product type in Kemiluppen.

Product category in Kemiluppen	Total products	MCI/MI or MI containing products (%)	Products containing MCI/MI	Products containing only MI
Facial care—cleansing/makeup remover/wash	1334	6 (0.45%)	6	0
Facial care—scrub/peeling	370	3 (0.81%)	1	2
Shaving and hair removal—shaving foam/gel/cream	249	1 (0.4%)	1	0
Shaving and hair removal—beard care	63	1 (1.59%)	1	0
Hair care—conditioner/treatment/mask	2293	98 (4.27%)	95	3
Hair care—hair dye	323	5 (1.55%)	5	0
**Hair care—shampoo**	**2784**	**127 (4.56%)**	**124**	**3**
Soap and hygiene—body wash	1613	7 (0.43%)	7	0
Soap and hygiene—bar soap	186	1 (0.76%)	1	0
**Soap and hygiene—liquid hand soap**	**1048**	**8 (0.76%)**	**8**	**0**
Total	10 263	257	249	8

*Note:* The two product categories selected for chemical analysis are highlighted in bold text. The percentage of each product type containing MCI/MI or MI is shown. The total products are the number of products present in Kemiluppen in each of the categories where some products contain MCI/MI or MI.

**FIGURE 2:**
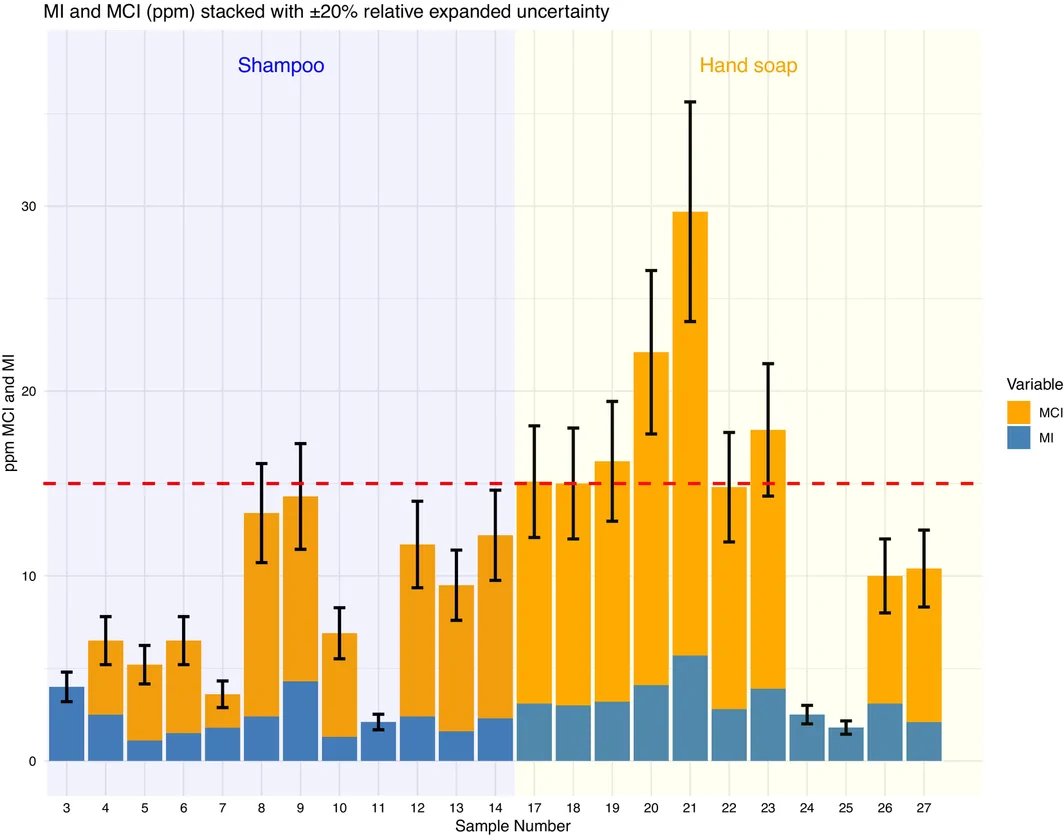
MCI and MI content stacked for each product. Only products with detected MCI or MI are shown. The graph is divided into shampoos (left, blue background) and hand soaps (right, yellow background). The red dashed line indicates the 15‐ppm limit set by European legislation for both MCI/MI and MI in rinse‐off products. Seven products did not have detectable levels of MCI/MI or MI and are not shown in this graph. Breaks in the sample numbering indicate a lack of detected MCI and MI (1–2, 15–16 and 28–30).

In total, 12/14 shampoos (sample 2–14) and all 11 hand soaps (sample 17–27) with declared MCI and/or MI contained detectable levels of MCI/MI or MI (Figure 2). Samples 1 and 2 (shampoos) did not contain detectable MCI/MI, despite having declared MCI/MI content. Two shampoos and two hand soaps only contained MI, which corresponded to the declared content. None of the two shampoos or three hand soaps with no declared MCI or MI content had any detectable levels of MCI or MI (Samples 1–2 and 28–30). All remaining products contained MCI/MI or MI as declared.

Two of the products were clearly above the regulatory limit of 15 ppm, taking the relative expanded uncertainty into account, containing a total of 30 and 22 MCI/MI, while four samples have a measured value of 15–18 ppm (Figure 2).

**FIGURE 3:**
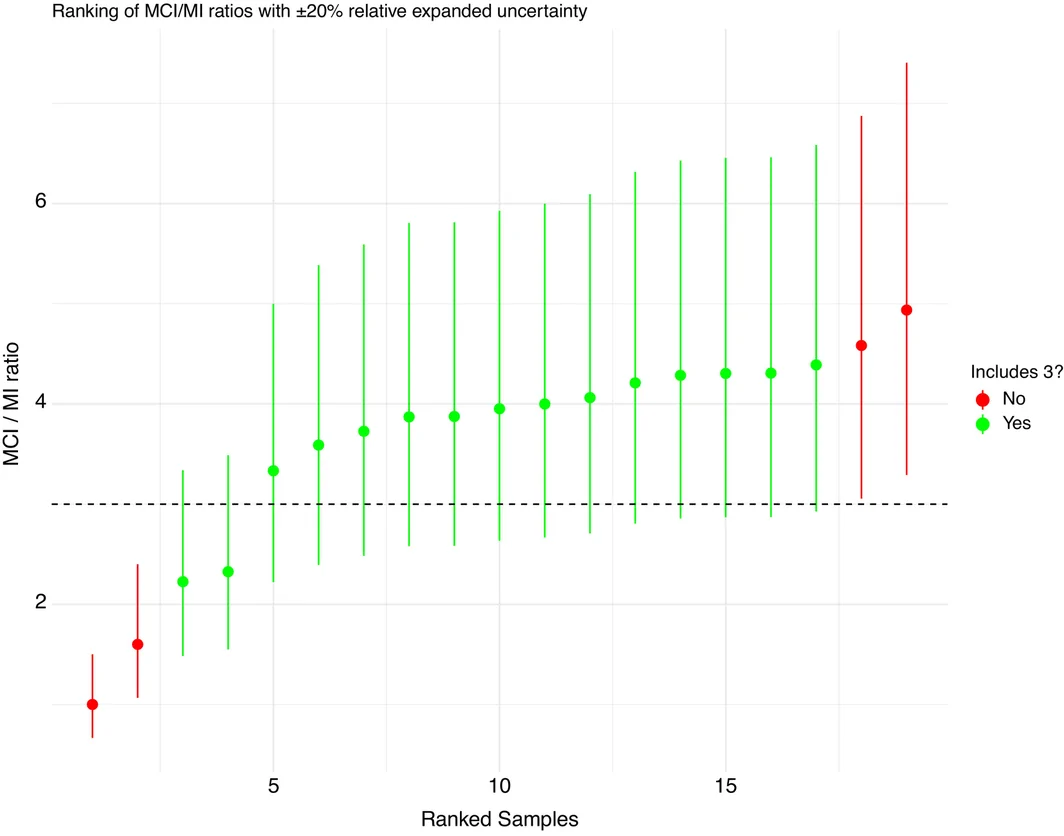
MCI/MI ratio ranges. Ratios are calculated by utilising ±20% relative expanded uncertainty. The samples are shown in a ranked manner meaning the sample numbers here do not correspond to the previous figure. The dashed horizontal line indicates the expected MCI:MI ratio of 3:1. The dots indicate the MCI/MI ratio from the measured values, while the lines indicate the range of possible ratios considering the combined standard uncertainty. Red colour: The ratio ranges do not include the possibility of a 3:1 ratio. Green colour: The ratio ranges include the possibility of a 3:1 ratio.

In Figure 3 the MCI:MI ratio for all products containing both MCI and MI is shown. The calculated MCI/MI ratio ranges show that four of the products do not include 3 as a possible ratio. This means that, given the ±20% uncertainty, it is not possible that the MCI/MI ratio is 3:1 (measured values ± and calculated ratios shown in Table 2), which is the only ratio permissible as per the European legislation [7].

**TABLE 2:** MCI and MI calculations of ratios for products not containing a possible ratio of 3.

MCI (±20%)	MI (±20%)	Measured ratio (MCI/MI)	Minimum ratio (MCI − 20%/MI + 20%)	Maximum ratio (MCI + 20%/MI − 20%)
1.8 (1.44–2.16)	1.8 (1.44–2.16)	1	0.7	1.5
4 (3.2–4.8)	2.5 (2–3)	1.6	1.1	2.4
11 (8.8–13.2)	2.4 (1.92–2.88)	4.6	3.1	6.9
7.9 (6.32–9.48)	1.6 (1.28–1.92)	4.9	3.2	7.4

*Note:* Measured values for MCI and MI ±20% were used for calculating the possible MCI/MI ratios. The calculation method for the measured, minimum and maximum ratios.

In five products where the online ingredient list and the physical ingredient list did not match, we found that while MCI and MI had been removed in the products, sodium benzoate had been added in three. The three preservatives present in the five non‐MCI/MI and MI products were benzyl benzoate, sodium benzoate and potassium sorbate (Table 3).

**TABLE 3:** Removed added and present preservatives.

Preservatives		
Removed (*n*)	Added (*n*)	Present (*n*)
DMDM Hydantoin (1) Sodium Benzoate (1) MCI (4) MI (5) Salicylic acid (2)	Sodium benzoate (3)	Benzyl benzoate (1) Sodium benzoate (4) Potassium sorbate (1)

*Note:* Aggregated list of removed, added and present preservatives in products (*n* = 5) where the online and physical ingredient list did not match (MCI/MI or MI declared online, but not on the physical product). Removed: Aggregated list of preservatives that have been removed from the products and therefore are no longer present on the ingredient lists. Added: Aggregated preservatives that are now part of the ingredients list but were not on the online declaration. Present: Aggregated list of preservatives currently present in the five products.

## 4 Discussion

To the best of our knowledge, this study represents one of the first larger‐scale investigations of MCI/MI and MI declaration in cosmetic and personal care products. Among the 10 263 products present in the categories with products containing MCI, 257 (2.5%) listed MCI and MI as ingredients. Shampoos and conditioners were the product categories most frequently containing MCI and/or MI, although the overall prevalence across all product types was low. Notably, several high‐use items, such as hand soaps, also listed MCI and MI among their ingredients.

A Danish market survey conducted in 2013, which examined a broad range of cosmetic products available in retail stores, reported that 3.3% of all products (60 of 1795) contained MI alone [16]. This contrasts with our findings, in which only eight products (< 0.1%) contained MI as the sole isothiazolinone preservative.

Several studies have examined the elicitation of allergic Contact Dermatitis following exposure to MCI/MI or MI. A dose–response repeat open application test (ROAT) conducted in 2011, designed to mimic real‐life use of leave‐on cosmetic products, demonstrated that concentrations as low as 5 ppm MI were sufficient to elicit dermatitis in MI‐sensitised individuals. A subsequent ROAT study from 2015 showed that 50 ppm MI elicited allergic Contact Dermatitis in 7 of 9 MI‐sensitised participants when applied as a liquid soap five times daily. The authors concluded that the true elicitation threshold for MI was considerably lower than 50 ppm [17].

MCI has been recognised as a more potent sensitizer than MI. According to an earlier opinion from the Scientific Committee on Cosmetic Products and Non‐Food Products (SCCNFP/0625/02), the lowest sensitising dose identified in Human Repeat Insult Patch Tests was 12.5 ppm for the MCI/MI mixture (3:1), compared with 400 ppm for MI alone [18]. Consequently, the presence and concentration of MCI in cosmetic formulations are of particular concern with respect to the induction of sensitisation [19].

Recent European studies have demonstrated that the proportion of MI‐sensitised patients who had used identified products containing MCI or MI linked to their Contact Dermatitis (i.e., clinical relevance) decreased from 73% in 2015 to 41.7% in 2022. This indicates a clear improvement, although concerns about ongoing exposure persist. Among patients with currently relevant MI contact allergy, rinse‐off cosmetic products are still the most frequently associated product type (73%) [8]. This may indicate that even the currently permitted levels in cosmetic products are too high, considering the few products containing MCI/MI or MI. However, in two products, the permitted limit of 15 ppm of the MCI/MI mixture and MI was exceeded (30 and 22 ppm). These two products were hand soaps, which is a critical product category, as they may be used multiple times a day for hand washing, in some scenarios and occupations more than 20 times daily as a standard [20]. It has been shown that the number of exposures is crucial for the risk of sensitisation [21, 22], as well as for elicitation of allergic Contact Dermatitis [23].

This apparent problem requires further investigation by looking into contents of hand soaps especially targeted for the work environment and expanding the number of products as in the current analysis only 8 hand soaps were identified. Interestingly, as seen in Figure 3, some products appear to have a different ratio than 3:1 for MCI/MI content, which is the only MCI/MI ratio accepted in the cosmetic regulation (Annex V entry 39) [7]. Other studies have similarly found that the MCI/MI ratios are not always 3:1 [24]. In the cosmetic regulation the entries for MCI/MI (entry 39) and MI (entry 57) are mutually exclusive, meaning a product may contain either MCI/MI (3:1) or MI [25]. The cause of the different ratios, whetherdue to chemical reactions of MCI or MI, other sources of MCI/MI, or imprecisions in the concentrate mixture obtained from the chemical supplier or by other possible factors is unknown. The ratio calculations were not formally investigated by the analytical laboratory and are therefore considered exploratory calculations. Further investigations are required to determine whether these results are reproducible. Nevertheless, since MCI is a stronger allergen than MI a skewed ratio in favour of MCI should be avoided to prevent sensitisation. In the two products with MCI/MI levels above the allowed limit, they also exhibited an MCI:MI ratio exceeding 3, showing that MCI was present in disproportionately higher amounts than MI, a trend that is also visualised in Figure 3.

Protection against MCI and MI sensitisation is not only important in a cosmetic context, as the use of MCI/MI has been steadily increasing both in tonnage and the number of products in non‐cosmetic products, while MI usage has remained constant as shown in a Swedish study covering the years 1995–2018 [26].

As seen by the decrease in MCI and MI contact allergy prevalence rates [27, 28] the restrictions laid out in the cosmetic regulation have had a significant and positive effect. Importantly, this decrease in the EU already began in 2013–2014 coinciding with the SCCS opinion on MI, which paved the way for the subsequent regulation [19].

As a risk management step all preservatives must be declared on cosmetic products in the EU (Article 19 in the cosmetic products regulation) [7]. During the purchase of products in this study, five soaps and shampoos were declared online as containing MCI/MI; however, when physically obtained, MCI/MI were no longer on the ingredient lists (confirmed by chemical analysis). This may be a result of the ongoing decrease in the use of MI in cosmetic products. However, the product information on their webpage had not been updated accordingly.

In this context, it is essential that regulatory or formulation changes do not trigger new ‘contact allergy epidemics’ related to preservative use. Among the five products in this study that demonstrated discrepancies between online and in‐store ingredient lists, sodium benzoate was the only preservative with a sensitisation profile of potential clinical importance (Table 3). A recent Swedish study reported a notable rate of positive patch test reactions to sodium benzoate in patients evaluated for cosmetic‐related skin problems, although the majority of reactions were mild [28]. Substituting one allergen with another may inadvertently introduce new contact sensitisers; however, few preservatives exhibit allergenic potency comparable to the isothiazolinones, particularly MCI [29]. Nevertheless, there is a clear need for more proactive approaches to risk assessment and management of skin sensitisation. The ongoing pattern of preservatives replacing one another in cosmetic formulations underscores this need [30]. Historically, timely risk management has proven difficult, yet individual patient cases and subsequent surveillance data often provide early warning signals of emerging allergenic hazards before they are fully acknowledged by industry stakeholders [31].

### 4.1 Limitations

The data in this paper are based solely on products scanned and uploaded to Kemiluppen. The observed frequencies may be influenced by selection bias driven by store inventories and user preferences; the app had more than 800 000 downloads as of 2024 [9] in a population of 6 million, however it cannot be regarded a random sample of products. The chemical analysis had a detection limit of 1 ppm, which is a satisfactory sensitivity.

All products were purchased in Denmark; however, as an EU member state, Denmark follows the same regulatory framework as all other member countries. Many products are also manufactured for broader regional markets, such as Scandinavia. Thus, concentrations exceeding the 15 ppm limit or deviations from the required 3:1 MCI/MI ratio may represent a wider European issue rather than a phenomenon specific to Denmark.

Furthermore, the relatively limited number of analysed samples compared to the vast and continuously evolving cosmetic market should be considered when interpreting the findings. In addition, products not declaring MCI/MI or MI were not included, and potential uncertainties in ingredient labelling may therefore not be fully captured, particularly in the global market.

In parallel, both MCI/MI and MI as a standalone preservative have seen a general increase in usage (tonnage) from 1995 to 2018 in non‐cosmetic products, for example as biocides for use in water‐based chemical products and mixtures [26]. It is a gap that these aspects have not been covered by the present investigation.

A further limitation is that only products declaring MCI/MI or MI were included in the chemical analyses. Consequently, the study does not address the potential presence of these preservatives in products without declared content, whether due to mislabelling, contamination or impurities in raw materials. Broader analytical screening of products irrespective of ingredient labelling would be needed to fully evaluate the reliability of ingredient lists.

### 4.2 Conclusion

MCI/MI and MI are still used in cosmetic products, although infrequently. All, except two products, complied with the regulatory limit. The two non‐compliant products were liquid hand soaps, a product category that, based on data from Kemiluppen, has very few products containing MCI/MI or MI. Given the continued occurrence of contact allergy, these findings may raise questions as to whether the current limit (15 ppm) is sufficiently protective, particularly in high‐frequency rinse‐off exposure scenarios. The use of these preservatives in rinse‐off cosmetic products, and their potential contribution to ongoing sensitisation, warrants further investigation. There remains a need for continued compliance monitoring, as well as more proactive approaches to risk assessment and management of skin sensitisation.

## Author Contributions

**Mathias Krogh Pedersen:** conceptualization, investigation, writing – original draft, methodology, writing – review and editing, formal analysis, data curation, visualization. **Jakob Ferløv Baselius Schwensen:** conceptualization, writing – review and editing, methodology, supervision. **Christel Søgaard Kirkeby:** writing – review and editing, resources. **Christina Rudén:** conceptualization, writing – review and editing. **Jeanne Duus Johansen:** conceptualization, writing – review and editing, methodology, project administration, supervision, funding acquisition. **Gianluca Selvestrel:** conceptualization, writing – review and editing. **Martin F. Wilks:** conceptualization, writing – review and editing. **Jose Hernán Alfonso:** conceptualization, writing – review and editing. **Stine Müller:** writing – review and editing, resources. **Steen Mollerup:** conceptualization, writing – review and editing.

## Funding

This work was supported by the European Union's Horizon Europe research and innovation programme (grant no. 101057014) and an unrestricted grant to The National Allergy Research center from the Danish Financial Act.

## Conflicts of Interest

Jose Hernán Alfonso has received an unrestricted research grant from Sanofi, honoraria for presentations from Sanofi, Almirall and Essity, and has served as an advisor for Leo Pharma and Novartis. Jakob Ferløv Baselius Schwensen has previously served as an investigator for Sanofi.

## Data Availability

The data that support the findings of this study are available from the corresponding author upon reasonable request.
